# The Utilization and Potential of Mindfulness-Based Stress Reduction Therapy in Individuals Diagnosed with Acute Coronary Syndrome

**DOI:** 10.31083/j.rcm2508277

**Published:** 2024-08-07

**Authors:** Jifa Tao, Xingkui Dou, Lixing Chen, Fei Hu, Zhengyu Li, Haipeng Gao, Xianbin Li, Min Zhang, Zhao Hu

**Affiliations:** ^1^Department of Geriatric Cardiology, The First Affiliated Hospital of Kunming Medical University, 650000 Kunming, Yunnan, China; ^2^Department of Cardiology, The Second Affiliated Hospital of Kunming Medical University, 650000 Kunming, Yunnan, China; ^3^Department of Cardiology, The First Affiliated Hospital of Kunming Medical University, 650000 Kunming, Yunnan, China

**Keywords:** mindfulness-based stress reduction (MBSR), acute coronary syndrome (ACS), percutaneous coronary intervention (PCI)

## Abstract

Acute coronary syndrome (ACS) poses a significant threat to health and well-being, although percutaneous coronary intervention (PCI) is an effective treatment method. However, many patients undergoing PCI for coronary heart disease often experience negative emotions such as depression and anxiety, as well as sleep disturbances, poor adherence to medications, and somatic symptoms. These adverse psychological effects can contribute to an increased risk of cardiovascular events. Mindfulness-based stress reduction (MBSR), a highly effective mind-body therapy, has been increasingly utilized in the recovery process of patients with coronary heart disease. Several scholars have conducted mindfulness interventions for post-PCI patients with coronary heart disease and achieved promising outcomes. This article primarily focuses on applying mindfulness-based stress reduction in PCI patients with coronary heart disease and its future prospects.

## 1. Introduction

Cardiovascular disease (CVD) is the most common cause of death and a serious threat to people’s lives and health [1]. In 2019, there were an estimated 5.8 million new cases of ischemic heart disease in the 57 Member States of the European Commission on Cardiology. Ischemic heart disease is the most common cause of CVD death, accounting for 38% of all CVD deaths in females and 44% in males [2]. Percutaneous coronary intervention (PCI) is an important treatment for coronary artery disease, especially acute ischemic heart disease. Early intervention to restore blood supply is essential for patients [3]. Some studies suggest that patients with coronary artery disease, particularly those diagnosed with acute coronary syndrome (ACS), are susceptible to experiencing negative emotions such as anxiety and depression, which may result in a decline in sleep quality, somatic symptoms, and poor adherence to medical advice [4, 5, 6]. Mindfulness-based stress reduction (MBSR) is a widely used psychotherapeutic training approach centered on mindfulness meditation [7]. It emphasizes present-moment attention while addressing the underlying causes of human suffering and ways to alleviate it [8, 9]. The MBSR program typically spans 8 weeks, encompassing one weekly session lasting approximately 2 hours. Experienced psychotherapists skillfully guided these sessions, which can be facilitated through diverse mediums such as online platforms or telephonic communication. During the initial half-hour of each session, participants receive comprehensive instruction on relevant concepts, followed by engaging in activities such as mindful breathing, seated meditation, mindful walking, body scans, gentle stretching exercises, and yoga to augment their focus on bodily sensations. To further cultivate individuals’ mindfulness awareness, they are encouraged to seamlessly integrate non-judgmental awareness into their daily lives as an effective coping mechanism for life’s stresses [10]. With the growing acceptance of the biopsychosocial model [11], MBSR has gained increasing attention and application within medical and educational fields.

## 2. Methods

The PubMed and CNKI databases were queried using the following broad search terms, including mindfulness, MBSR, ACS, coronary heart disease, PCI, depression anxiety, somatic symptoms, adherence, and insomnia from January 2000 to February 2023. All preclinical (*in vitro* and animal) and clinical studies (randomized, non-randomized, prospective, retrospective) were deemed suitable except for case studies. Supplementary papers that could be relevant for inclusion were identified by cross-referencing the articles’ bibliography.

## 3. The Advantages of Mindfulness-Based Stress Reduction Therapy in Patients Following Percutaneous Coronary Intervention for Coronary Artery Disease

### 3.1 The Intervention Contributes to the Amelioration of Adverse Affective States, such as Anxiety and Depression

One study [12] revealed that a considerable proportion of patients, up to 40%, experience notable levels of depression, anxiety, and, thus, stress [13] following an acute myocardial infarction or other serious cardiac event or surgery. These negative emotions can have detrimental effects on mental health and pose challenges and obstacles to patients’ behaviors and lifestyles, potentially leading to increased healthcare costs as well as heightening risks of mortality and other adverse outcomes. A meta-analysis demonstrated no significant disparity in the efficacy between pharmacotherapy and individualized psychotherapy for depression and anxiety disorders in the general population. However, pharmacotherapy exhibits limited effectiveness in cardiac patients without improving long-term prognosis [14]. Milan R van Dijk *et al*. [15] conducted a study involving 1411 eligible patients who reached stability 6 months postoperatively after undergoing PCI at Erasmus Medical Center in Rotterdam. They prospectively followed up with all participants, ultimately obtaining data from 1112 individuals (78.8%) who completed the Hospital Anxiety and Depression Scale assessment. The findings indicated that depression was independently associated with a 77% increased risk of all-cause mortality ten years after PCI, regardless of the presence of anxiety [15]. On the other hand, MBSR relaxation enhances negative emotion management [16] while promoting cardiac rehabilitation and reducing the incidence of adverse cardiovascular events [17]. Nijjar *et al*. [12] allocated patients who underwent coronary heart disease PCI to either an 8-week MBSR intervention group or a usual care group in a ratio of 2:1 (intervention:control). Data on depression and anxiety were collected at follow-up after randomization, with over 87% of MBSR patients completing the intervention, and study retention exceeded 95% at each follow-up visit. At the three-month mark, there was a significant improvement in depression and anxiety among MBSR patients compared to the control group. This pilot randomized controlled trial (RCT) provides preliminary evidence of the potential for MBSR to improve short-term psychosocial well-being in cardiac patients during their first year of recovery. MBSR has a positive effect on stress management. James A Blumenthal *et al*. [18] conducted a study in which 151 patients with coronary heart disease (CHD) aged 36 to 84 years were randomized to comprehensive cardiac rehabilitation (CR) or comprehensive CR combined with stress management training (SMT) (CR + SMT) for 12 weeks. All participants were followed for clinical events up to 5.3 years (median, 3.2 years). The results showed that patients randomized to CR + SMT showed a significant reduction in the level of composite stress compared to patients who received comprehensive CR alone (*p* = 0.022), which was mainly driven by improvements in anxiety, distress, and perceived stress [18]. Ye Mengsi *et al*. [19] selected 200 coronary heart disease PCI patients as research subjects and conducted positive thinking interventions on the experimental group, finding that their anxiety scores and depression scores were significantly lower than in the control group, demonstrating that positive thinking stress reduction therapy can alleviate negative emotions such as depression and anxiety experienced by post-coronary heart disease PCI patients. Many studies have confirmed the short-term therapeutic efficacy of MBSR in mitigating negative emotions. However, its long-term effects remain uncertain, potentially attributed to the sustainability of MBSR interventions. Specifically, numerous studies have conducted follow-up assessments on participants from the experimental group after an initial 8-week mindfulness intervention. Nevertheless, subsequent surveys failed to ascertain whether participants maintained regular self-practice.

### 3.2 Contributes to the Enhancement of Sleep Quality

Coronary heart disease patients primarily consist of elderly individuals, and the prevalence of sleep disorders tends to increase with age. Among the elderly population, insomnia is prevalent in approximately 30%–48% of cases, characterized by reduced total sleep time, sleep efficiency, and deep sleep. Insomnia severity is associated with overall health and quality of life, as well as hypertension, myocardial infarction, and potentially stroke [20]. Therefore, it is crucial to prioritize improving patients’ sleep when developing rehabilitation strategies for PCI patients with coronary artery disease. Currently, available approaches for addressing sleep disorders mainly involve benzodiazepines or psychotherapy. Benzodiazepines can reduce the time taken to fall asleep and increase total sleep duration within two weeks; however, they come at the expense of decreased rapid eye movement (REM) sleep and the duration of deep sleep [21]. Long-term use of benzodiazepines is linked to an increased risk of dementia; hence, its prolonged usage is not recommended. A recent systematic review analyzing observational studies revealed that long-term users had a 1.5- to 2-fold higher risk of dementia compared to non-users [22]. Since psychological methods are prone to generating long-lasting advantages without the potential for tolerance or negative consequences linked to pharmacological methods, cognitive behavioral therapy for insomnia (CBT-i) is frequently suggested as the initial course of action in addressing chronic insomnia [23]. The positive thought intervention for insomnia treatment was initially derived from Kabat-Zinn’s MBSR program. Mindfulness-based stress reduction courses aim to educate individuals on directing their attention inward using various meditation techniques. Participants are instructed to observe their immediate emotions and physical state without attempting to alter, suppress, or evaluate thoughts. By engaging in mindfulness training, participants acquire the ability to perceive their thoughts as mental occurrences rather than truths, thereby avoiding excessive anxiety about the future or dwelling on past concerns. By “breaking” the cycle of rumination and worry, it promotes the disengagement necessary for sleep [24]. A clinical randomized controlled trial conducted by Gross *et al*. [25] compared the efficacy of MBSR with medication in treating primary chronic insomnia. Thirty adults were randomly assigned to either an 8-week MBSR group or a medication group receiving nighttime dexzopipramone intervention in Pittsburgh, Pennsylvania. The results showed that both groups experienced improvements in their Pittsburgh Sleep Quality Index, Insomnia Severity Index, and sleep self-efficacy after the intervention. These findings suggest that MBSR is a viable and effective treatment option for individuals with chronic insomnia. The study by Fan J and Yang J [26] involved a sample of 196 post-PCI patients, wherein the experimental group received the intervention of positive thinking. The results indicated significant improvements in various sleep quality scores and the overall score compared to the control group. Therefore, it can be concluded that MBSR is associated with better sleep quality among post-PCI patients with coronary artery disease.

### 3.3 Reinforcing Patient Adherence

Significant advancements in pharmacology have provided crucial support for reducing morbidity and mortality associated with cardiovascular disease. However, non-adherence to prescribed medications remains a significant challenge in improving patient outcomes. For patients undergoing PCI for coronary heart disease, long-term utilization of anti-platelet drugs and statins is imperative. However, long-term medication, potential side effects, age-related factors, and financial constraints may be associated with a decrease in patient adherence to medical advice, which could potentially impact their recovery process [27]. In a study conducted by Liu D and Dong S [28], 120 post-PCI coronary artery disease patients were randomly divided into an observation group (conventional intervention + MBSR; n = 60) and a control group (conventional intervention; n = 60). A comparison of the medication adherence between these two groups found that the observation group exhibited significantly better adherence than the control group.

### 3.4 Assist in Alleviating Symptoms of Somatization

Patients who have undergone PCI for coronary artery disease often experience a wide range of somatization symptoms, including chest pain, chest tightness, and dyspnea. These persistent somatization symptoms pose a serious threat to a patient’s health, with affective disorders and anxiety disorders being the most common complications. Due to the subjective nature of the patient’s experience and the lack of objective evidence for organic disorder, it can be challenging to address these symptoms during consultations [29]. Charlotte Dewsaran-van der Ven *et al*. [30] administered Self-Compassion Scale (SCS), number of symptoms (PSC), and health-related quality of life (EQ-5D) questionnaires to 236 patients diagnosed with somatoform disorders, along with an age- and sex-matched control group from the general population. The results revealed significant disparities in self-compassion levels between patients with somatoform disorders and the general population, demonstrating a moderate effect size (d = –0.65). Multiple regression analyses indicated that both somatoform disorders and low self-compassion were independently associated with symptom severity and diminished health-related quality of life. These findings suggest that self-compassion is crucial in treatment outcomes for individuals with somatoform disorders, highlighting its potential as a targeted intervention. The MBSR program can effectively help patients redirect their attention to the present moment and enhance their perceptions of the surrounding environment. Additionally, it can foster an increased level of self-compassion by encouraging non-judgmental awareness towards these perceptions, ultimately leading to improvements in somatic symptoms [30].

## 4. Possible Mechanisms

### 4.1 The Implementation of Mindfulness-Based Stress Reduction Contributes to the Enhancement of Autonomic Nervous System Regulation

In patients who have undergone PCI for coronary artery disease, autonomic nervous system dysfunction is associated with corticotropin-releasing factor (CRF) dysfunction, which may lead to an abnormal adrenocorticotropic hormone (ACTH) response [31]. This is associated with increased cortisol levels, increased catecholamine release, increased cardiac output and oxygen consumption, increased heart rate, decreased heart rate variability, and increased sympathetic tone [32], which increases cardiac workload and decreases peripheral vascular resistance. The augmented myocardial oxygen consumption is accompanied by a decrease in coronary blood supply, leading to an imbalance between supply and demand that may result in myocardial ischemia. MBSR regulates autonomic function, mitigates hormone release disorders, improves patients’ blood pressure and heart rate, and reduces myocardial ischemia [33]. Heart rate variability (HRV) is a well-established and validated method for assessing the sympathetic-vagal balance of the heart; reduced HRV serves as a poor prognostic indicator for life-threatening arrhythmia in patients with myocardial infarction and heart failure. Mindfulness-based stress reduction over 8 weeks utilizes standardized techniques that enable patients to engage in breathing exercises and emotional calmness, which aid in improving autonomic regulation and enhancing cardiac sympathovagal balance [34, 35].

### 4.2 The Implementation of Mindfulness-Based Stress Reduction Facilitates Effective Stress Management 

Stress is closely linked to myocardial ischemia, and patients undergoing PCI for coronary artery disease encounter various stressors, including physical discomfort, significant financial burden, and poor interpersonal relationships, among others. These stressors often exert a detrimental impact on the long-term cardiac recovery of patients. Acute and intense emotional conditions, which themselves are major factors of stress, can, thus, result in stress-induced cardiomyopathy characterized by heart changes resembling an “octopus bottle” and clinical symptoms similar to acute myocardial infarction. Chronic stress frequently accompanies alterations in autonomic function as well as dysfunctions in the neuroendocrine system, immune system, cognitive function, and behavioral patterns that contribute to cardiovascular disease development. Evidence suggests that incorporating stress management training into cardiac rehabilitation programs effectively mitigates patient’s stress levels, reduces adverse cardiovascular event incidence, significantly improves prognosis, and enhances quality of life [18]. Mindfulness-based stress reduction enables participants to focus on the present moment without judgment or attachment towards what is happening around them while cultivating inner peace and adopting an open-minded attitude towards life for better coping with stressful situations.

### 4.3 The Implementation of Mindfulness-Based Stress Reduction Contributes to the Enhancement of Endothelial Function

The concept of the endothelial injury theory is a fundamental cornerstone in the field of atherosclerosis due to the pivotal role played by the endothelium in maintaining vascular homeostasis and ensuring normal circulatory function through the release of contractile and diastolic factors. Furthermore, it significantly contributes to hemostasis, inflammation, and metabolism [36]. Patients with coronary artery disease often experience impaired endothelial function and microcirculatory disorders due to chronic or acute stresses that result in the inhibition of nitric oxide release by glucocorticoids, endothelin-1, proinflammatory mediators, activation of endothelin A receptors, among other issues [37, 38]. Mindfulness-based stress reduction can effectively enhance endothelial function by encouraging patients to focus on self-awareness regarding their physical and mental well-being while maintaining a state of calmness.

### 4.4 The Application of Mindfulness-Based Stress Reduction Contributes to the Enhancement of Epigenetic Mechanisms

Epigenetic expression serves as a crucial link between phenotypic manifestation and disease susceptibility. Additionally, an individual’s epigenomic and epigenetic characteristics influence their behavior, stress response, disease vulnerability, and even lifespan [39]. Notably, both factors are potentially malleable and can impact gene expression through lifestyle and environmental elements such as nutrition, exercise, behavioral modifications, and positive stress reduction techniques [40, 41]. In primates, heightened overall DNA methylation has been associated with amplified behavioral stress responses following early life stress [42]. Mind-body therapies such as positive stress reduction practices, meditation, and yoga are emerging as highly promising interventions for altering one’s mindset and lifestyle while reducing risk factors for coronary heart disease.

### 4.5 The Mindfulness-Based Stress Reduction Elicits Neuroplastic Changes in the Brain

A study by Lenhart L *et al*. [43] discovered that stress reduction interventions utilizing positive thinking induced a wide range of dynamic changes in the brain. In their study, Nakamura H *et al*. [44] developed a functional model of the brain based on anatomical networks and a computational model representing information propagation between brain regions by simulating activity changes in each region. They investigated the impact of positive thinking on information propagation within the brain, with simulated changes reflecting the resource allocation to neural activity by adjusting the output weights of each region. The simulations revealed that therapy focused on reducing stress through positive thinking decreases excitability in cortical regions associated with negative emotions, such as the amygdala. Hölzel BK *et al*. [45] demonstrated that gray matter regions in participants’ brains increase due to training aimed at promoting positive thoughts, highlighting the modifiability of the nervous system. Alsubaie *et al*.’s findings [46] indicated that individuals who practiced MBSR exhibited improvements in social anxiety symptoms compared to those who engaged in aerobic exercise alone, as evidenced by self-referenced brain network correlations. Functional magnetic resonance imaging and results from self-referential coding tasks showed significant alterations in self-perception and dorsomedial prefrontal cortex activity among MBSR practitioners, which were associated with notable reductions in social anxiety [46]. Pedro Morais *et al*.’s [47] research revealed that MBSR can influence an individual’s emotional state and attention level according to physiological indicators from both the autonomic and central nervous systems.

## 5. Conclusions 

MBSR places greater emphasis on the participants and focuses more on the psychological aspects of health. Cardiac rehabilitation based on this therapy is characterized by its low cost, low risk, effectiveness, convenience, and ease of implementation, offering numerous advantages. Consequently, it has gained acceptance among increasing individuals and is widely utilized across various fields. However, despite its widespread use, the underlying mechanism must be clarified, necessitating further clinical and fundamental research efforts.
